# Is wearing a pedometer associated with higher physical activity among adolescents?

**DOI:** 10.1016/j.ypmed.2013.01.015

**Published:** 2013-05

**Authors:** Vanda Ho, Rebecca K. Simmons, Charlotte L. Ridgway, Esther M.F. van Sluijs, Diane J. Bamber, Ian M. Goodyer, Valerie J. Dunn, Ulf Ekelund, Kirsten Corder

**Affiliations:** a Murray Edwards College, Huntingdon Road, Cambridge, CB3 0DF, United Kingdom; b MRC Epidemiology Unit, Institute of Metabolic Science, Box 285, Addenbrooke's Hospital, Hills Road, Cambridge, CB2 0QQ, UK; c UKCRC Centre for Diet and Activity Research (CEDAR), Box 296, Institute of Public Health, Forvie Site, Robinson Way, Cambridge, CB2 0SR, UK; d Developmental Psychiatry Section, Department of Psychiatry, University of Cambridge, Douglas House, 18b Trumpington Road, Cambridge, CB2 8AH, UK; e Department of Sports Medicine, Norwegian School of Sports Sciences, PO Box 4014, Ullevål Stadion, 0806 Oslo, Norway

**Keywords:** Adolescent, Physical activity, Self-monitoring, Pedometer, Reactivity

## Abstract

**Objective:**

To examine whether wearing a pedometer was associated with higher objectively-measured physical activity (PA) among adolescents independent of other behavior change strategies, and whether this association differed by sex or day of wear.

**Method:**

In a parallel-group population-based cohort study, 892 adolescents (43.4% male, mean ± SD age, 14.5 ± 0.5 years) from Eastern England were recruited. PA was measured (in 2005–2006) by accelerometry over four days; a sub-group (n = 345) wore a pedometer coterminously with the accelerometer. Three-level (individual, day of wear and school level) multiple linear regression was used to examine the association between accelerometry (counts/min, cpm) and pedometer wear, stratified by sex and adjusted for weekday/weekend.

**Results:**

For the entire cohort, there was a significant decline in cpm over four days (p < 0.01). Girls wearing pedometers had higher mean cpm than those not wearing a pedometer, independent of BMI z-score, socio-economic status, weekday/weekend, and school clustering (β = 5.1; 95% CI: 0.8 to 9.5, p = 0.02). This association was not seen in boys.

**Conclusion:**

Pedometer wear was associated with higher PA among adolescent girls, but not boys. Findings may support sex-specific intervention strategies. In addition to pedometer monitoring, additional strategies may be required to promote PA levels, especially among boys.

## Highlights

► Pedometer wear was associated with higher PA among adolescent girls, but not boys. ► Findings may support sex-specific intervention strategies. ► Pedometer monitoring alone may not be sufficient to promote adolescent PA.

## Introduction

Insufficient physical activity (PA) among young people is associated with increased risk of obesity and related metabolic disorders (Ekelund et al., 2012). Rapid decreases in PA occur during adolescence (Dumith et al., 2011) and track into adulthood (Telama et al., 2005). Thus, adolescence is a critical period for promoting PA (Corder et al., 2010).

Adolescent PA promotion interventions are often ineffective (Camacho-Miñano et al., 2011; Kriemler et al., 2011; van Sluijs et al., 2011); the reasons for this are largely unknown. One possibility is that adolescents are not aware that they are insufficiently active and therefore are not susceptible to PA promotion (Corder et al., 2011). A promising avenue of PA promotion among adolescents is self-monitoring of behavior (Michie et al., 2009) which may improve awareness of (inadequate) PA levels (Corder et al., 2011).

Pedometers are low cost and provide an interpretable estimate of steps taken with potential for wide scale public health use (Tudor-Locke et al., 2011). Pedometers are often used for self-monitoring PA (Lubans et al., 2009) and as motivational tools (Kang et al., 2009). A recent review examining self-monitoring using pedometers in PA promotion reported a positive association in 3 of the 5 studies among adolescents (Lubans et al., 2009); most included studies combining multiple behavior change strategies together with self-monitoring. Among a small number of young adolescents, viewing pedometer step counts was associated with more steps/day compared to a sealed condition (Shimon and Petlichkoff, 2009), although this study did not use an objective measure to quantify PA. To our knowledge, there is little evidence of how recording pedometer step counts among a large population-based sample of adolescents is associated with objective PA independent of other behavior change strategies.

We hypothesize that wearing a pedometer and recording steps may increase adolescent awareness of their physical activity and therefore be associated with physical activity levels between groups who are wearing and not wearing a pedometer. In the current study we examined whether wearing a pedometer was associated with higher objectively-measured PA and whether this association differed by sex or day of wear.

## Methods

In the ROOTS study (Goodyer et al., 2010), adolescents from Cambridgeshire and Suffolk schools, UK were recruited at age 14 years to be reassessed with repeat measures of PA and mental health. Between April 2005 and December 2006, 27 secondary schools were approached, and 18 agreed to participate. Study information and consent forms were sent to parents of children aged 14 via schools. Body composition and PA were assessed between November 2005 and July 2007. Of 1185 participants originally agreeing to participate, 998 adolescents and parents (84%) completed postal assent and informed consent for the PA assessment and 931 (93%) attended a school measurement session. The ROOTS study was approved by the Cambridge Local Research Ethics Committee.

## Physical activity measurement

Free-living PA was assessed in 931 participants using the Actiheart combined heart rate and movement sensor (CamNTech, UK). The monitor which has been validated to assess PA in youth (Corder et al., 2005, 2007), is attached to the torso using two standard electrocardiogram electrodes and collects heart rate data and includes a uniaxial accelerometer. Data were recorded in 30 s epochs and participants were instructed to wear this waterproof monitor continuously for 24 h, for the remainder of the testing day and then four consecutive days, including a weekend. Participants were fitted with the monitors at the measurement session, and asked to return them one week later.

Overall PA was expressed as mean counts per minute (cpm) for each day of wear. Only movement data were used as we wanted to capture all movement including light-intensity PA; for which heart rate monitoring may have accuracy-related limitations (Livingstone et al., 2000). A custom program removed data recorded after 11 pm and before 6 am; periods of ≥ 60 min with continuous zero counts and days with < 600 min of recording. To derive time spent in moderate and vigorous PA (MVPA) accelerometry data were transformed for comparability with the Actigraph (Actigraph counts = Actiheart counts × 5) (Ridgway et al., 2011). Time (min/d) in MVPA was derived using 2000 (Actigraph) cpm on the transformed data as the lower threshold which has been used previously (Riddoch et al., 2004) and is equivalent to walking at 4 km/h (Ekelund et al., 2003). Eight participants had incomplete anthropometric data, leaving 892 individuals with accelerometry data.

## Pedometers

A sub-group (n = 376) wore a pedometer (OMRON HJ-109) coterminously with the accelerometer. Of those, 373 participants provided at least one day of pedometer data. Subgroups were selected opportunistically on a block basis; if there were enough pedometers for every participant in a measurement session then participants were given pedometers. A mean(SD) of 43.1(23.5)% of students per school wore a pedometer. This pedometer model demonstrates good inter-reliability between units and accuracy of less than 5% error at walking speeds above 1.56 m/s (Baker et al., 2010; Ryan et al., 2006). Participants were fitted with both monitors in the same measurement session. Pedometers were worn at the waist for four consecutive days, starting the day after the measurement session, and were taken off during sleep and water-based activities. Participants were given pedometer diaries and asked to record the following for each day of wear: the time the pedometer was put on and taken off, the duration of time the pedometer was off during the day (if any), and the total step count when the pedometer was removed at night.

Daily pedometer wear time was calculated by subtracting any time removed during the day from total wear time. If the pedometer was taken off for less than 10 min during a single day, this was ignored. Pedometer and accelerometer data were synchronized by date, participants with at least one matched day of data were included in the final analysis in the pedometer group (n = 345). The decision to include one day of data was based on pedometer data from 11,669 5- to 19-yr-olds where the first measurement day provided good reliability relative to the whole week (ICC = 0.79) (Craig et al., 2010).

## Biological and social variables

Age and sex were self-reported. Height was measured to the nearest 0.1 cm (Leicester Height Meter; Invicta Plastics, Leicester, UK). Weight was measured to the nearest 0.1 kg (TBF-300A; Tanita, Tokyo, Japan) in light clothing without shoes and socks. BMI was calculated and z-scores derived (Cole, 1997). Previously validated and published equations were used to derive body fat percentage (Tyrrell et al., 2001). Waist circumference was measured horizontally 1 cm above the umbilicus using a fiberglass d-loop tape measure. The ‘A classification of residential neighborhoods’ index was used as a proxy for area-level socio-economic status, which categorizes UK postcodes into one of five categories using 125 demographic and 287 lifestyle variables (ACORN). Data are presented in three categories: high SES (wealthy achievers, urban prosperity), middle SES (comfortably off) and low SES (moderate means and hard-pressed). Where postcodes are missing and SES data were unavailable (n = 10), the school mean was used.

## Statistical analysis

Analyses were carried out using STATA 11.0 (Statacorp, College Station, TX, USA). Participant characteristics were described by sex and for participants with and without pedometer data. Data were summarized using means and standard deviations for normally distributed variables and medians and inter-quartile ranges for skewed data. Students T-tests were used to compare characteristics between pedometer groups, stratified for sex. Tests for trend (Wilcoxon rank sum test (Cuzick, 1985)) were used to examine patterns of mean daily accelerometry counts per minute (cpm) over the four days of wear separately by sex and by pedometer wear. Three-level multiple linear regression (day of wear, child and school level) was used to quantify the association between accelerometry cpm and pedometer status over all days of wear. All models were run separately by sex and adjusted for BMI z-score, SES, weekend/weekday and school-level clustering. Wald statistics were used to assess model fit. The reliability of MVPA per participant when including one and four days of data was examined using large one-way ANOVAs. A sensitivity analysis was done removing the 11 participants with only one day of matched pedometer and accelerometer data and results were not materially changed.

## Results

Descriptive characteristics are summarized in Table 1. At least one day of complete date-matched pedometer and accelerometry data was available for 892 adolescents and 345 wore a pedometer. No significant differences in MVPA (p = 0.50), BMI (p = 0.88) or SES (p = 0.93) were found between participants who wore pedometers and those who did not. Reliability of MVPA did not markedly differ according to the number of days of matched pedometer and accelerometer data: > 1 day r = 0.78 and 4 days r = 0.80.

**Fig. 1:**
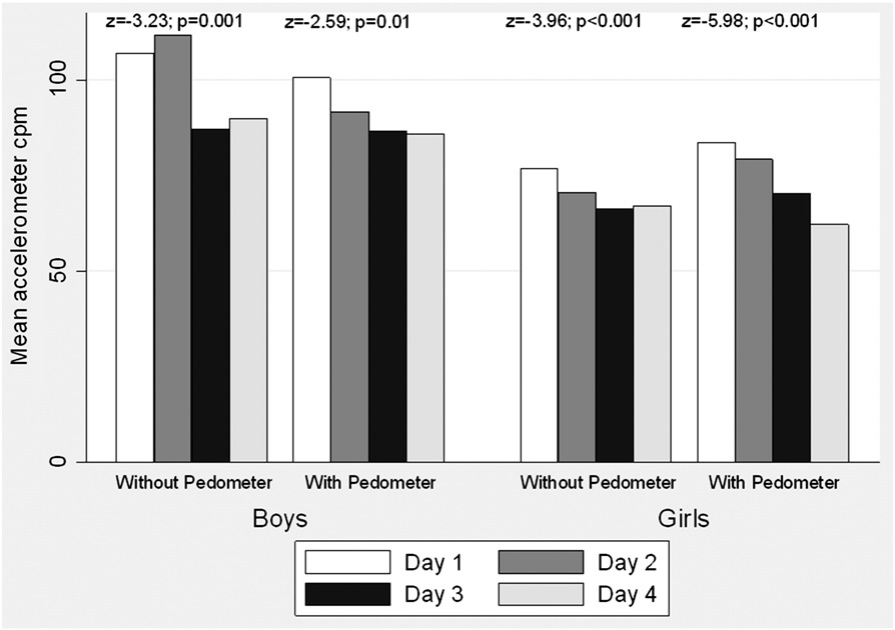
Mean accelerometry (cpm) by day of wear, separately by pedometer group and sex. p values are unadjusted tests for trend across days of wear within subgroups of adolescents (43.4% male, mean ± SD age, 14.5 ± 0.5 years) from November 2005 to July 2007 in Cambridgeshire and Suffolk, Eastern England. The z and p values are from Wilcoxon rank sum tests with correction for ties.

Mean counts per minute (cpm) by day of wear for pedometer-wear groups are shown separately by sex in Fig. 1 with tests for trend showing PA declines for each group over the four days of measurement. Compared to weekdays, weekend PA (88.4 vs. 75.6 cpm respectively, p < 0.001) and MVPA (55.9 vs. 44.1 min respectively, p < 0.001) were lower.

In the pedometer group, boys accumulated more steps than girls (Table 1). Among boys, overall mean accelerometer cpm did not differ by pedometer wear. Girls who wore a pedometer had higher overall mean accelerometer cpm than girls who did not wear a pedometer (Table 1).

Results from three-level multiple linear regression assessing PA (accelerometry cpm and MVPA minutes/day) adjusted for BMI z-score, weekend/weekday and SES are shown in Table 2. Accelerometry cpm over all days of measurement was not significantly different between boys with and without pedometer data. Among girls, there was a significant association between accelerometry cpm and pedometer use, with higher accelerometer cpm over all four days of measurement among girls wearing a pedometer. These associations were not significant for MVPA unadjusted for wear time for either boys or girls.

## Discussion

Among boys, overall mean accelerometer cpm did not differ by pedometer wear but girls who wore a pedometer had higher PA than those not wearing a pedometer. For both girls and boys, there was a significant decline in PA over four days of monitor wear. Although any higher PA associated with pedometer wear appears to be short lived, and restricted to girls, these results suggest pedometers may have promise for health promotion among adolescent girls.

For both boys and girls, PA declined stepwise over four days of wear for groups with and without pedometers. Viewing step counts was associated with more steps/day compared to a sealed control condition among a small number of young adolescents (Shimon and Petlichkoff, 2009) although this study did not use an objective measure to quantify PA. PA data were expressed as average counts per minute acting as adjustment for any differential wear time. Although the decline in accelerometer cpm over the four days of measurement is likely to be partly due to a drop in PA during weekends, the association remained when adjusted for weekday/weekend. This stepwise drop in PA could be due to participants adapting their behavior in response to measurement as this decline was observed for all participants in both pedometer and non-pedometer groups and is virtually unavoidable with objective PA measurement (van Sluijs et al., 2006). To our knowledge, this reactivity has not been clearly shown using objective PA measurement among adolescents and should perhaps be taken into account when measuring short term PA. Researchers did not specifically inform participants that the chest mounted accelerometer was measuring their PA but they were aware that their heart rate was being monitored. It is possible that participants were initially keen to increase their PA, with the novelty subsiding on a daily basis, declining to the same level as the non-pedometer group by the fourth and final day.

For girls, there were higher overall PA levels and a greater stepwise decline in accelerometer cpm in the pedometer group compared to the non-pedometer group. There were no differences in MVPA unadjusted for wear time in the final model but this could be due to lower absolute MVPA during the first day of wear as monitors were fitted during school. Our results may suggest that the combined measurement effect of both the accelerometer and a pedometer is perhaps greater than that of an accelerometer alone for adolescent girls. The accelerometer was a ‘black box’ with no visible activity output, placed on the torso and not removed throughout the study, while pedometer step counts were recorded in a diary. Participants may therefore be more conscious of the pedometer and it may be more likely to encourage them to be active. A recent review of pedometers used as motivational tools concluded that studies involving only female participants had a greater effect than when involving males (Kang et al., 2009). It is possible that this association may be stronger as social desirability bias appears more prevalent for girls, as seen with dietary reports (Hebert et al., 1997).

Graphing of step counts by young adolescents is associated with greater increases in step counts than when participants view (but do not graph) their step counts (Shimon and Petlichkoff, 2009). Research investigating the most effective way to utilise step count data to encourage behaviour change including refining different feedback methods and quantifying their effect on PA would be valuable (Watkinson et al., 2010).

To our knowledge, this is the first study to examine measurement effect of pedometer wear among a large adolescent population based sample assessed using objective PA. Attendance to the PA assessment visit was high (93%) but the high socio-economic background of participants (64%) and mostly normal BMI z-scores (83%) are a limitation. We cannot discern whether PA on the final day of measurement is raised compared to ‘true levels’ as we have no PA measure prior to study participation. We are unable to determine how long this measurement effect may last which may be relevant as a previous study among 9–12 year-olds found a gradual step count decline step counts occurring over 3 weeks (Ling et al., 2011). Choice of participants to wear pedometers was not systematic and could have led to selection bias, although no differences in MVPA, BMI or SES were seen between groups. We used accelerometry cpm (a measure of activity intensity) to examine effects of wearing a pedometer (which assesses activity volume). This was considered appropriate considering the significant correlation between step counts and accelerometry cpm (r = 0.47, p < 0.001). Participants wore accelerometers but not pedometers while swimming. We do not know if or when participants swam and are therefore unable to remove the associated accelerometer data. However, uniaxial accelerometer counts should be minimal during swimming. The pedometer group was not subdivided to wear sealed and open pedometers given the post-hoc nature of this analysis. Further research, including a randomized controlled trial would be necessary to confirm or refute our findings.

## Implications

A positive association between PA and pedometer wear was observed among girls but not boys. These results may provide preliminary evidence that pedometers could be useful tools for short-term PA promotion in adolescent girls. In addition to pedometer monitoring, additional complementary strategies such as tailored step goals, may be required in PA promotion among adolescents.

## Conflict of interest statement

The authors declare that there are no conflicts of interests.

## Figures and Tables

**Table 1:** Characteristics of the sample (43.4% male, mean ± SD age, 14.5 ± 0.5 years from November 2005 and July 2007 in Cambridgeshire and Suffolk, Eastern England) by sex and by pedometer measurement.

Variable^a^	With pedometer measurement		Without pedometer measurement	
Boys (n = 154)	Girls (n = 191)	Boys (n = 236)	Girls (n = 311)
Age, years	14.4 (0.5)	14.5 (0.5)	14.5 (0.5)	14.5 (0.5)
Socio-economic status, n (%)				
High	64.3	66.0	64.0	62.4
Middle	22.1	22.5	21.6	24.1
Low	13.6	11.5	14.4	13.5
Body Mass Index, kg/m^2^	20.2 (3.2)	21.1 (3.3)	20.4 (3.4)	21.1 (3.6)
Overweight (%)	13.6	17.8	14.4	14.5
Normal (%)	86.4	82.2	85.6	85.5
Accelerometry cpm	92.5 (37.3)	75.5 (28.9)	92.4 (32.0)	70.0 (22.5) b
Weekday accelerometry cpm	103.0 (43.8)	79.4 (29.4)	104.0 (52.2)	75.6 (25.0)
Weekend accelerometry cpm	80.4 (73.1)	70.1 (40.4)	80.0 (36.8)	63.8 (29.2) b
MVPA, minutes/day	58.6 (32.2)	43.5 (23.5)	61.9 (41.6)	41.5 (20.2)
Weekday MVPA, minutes/day	67.2 (37.1)	47.1 (27.0)	67.3 (33.9)	46.9 (24.2)
Weekend MVPA, minutes/day	48.7 (43.3)	41.7 (40.9)	54.1 (71.0)	35.7 (30.7)
Number of pedometer steps/day c	11,507 (7421)	10,393 (5409)		
Waist circumference (cm)	76.3 (8.4)	74.4 (8.6)	74.9 (8.4)	74.3 (8.8)
Body fat %	12.2 (6.5)	25.9 (7.2)	12.2 (6.8)	24.8 (7.9)

a All data are mean (SD) unless otherwise indicated.

b Significant difference between groups with and without pedometer data.

c Values are median (IQR) as data are skewed. MVPA = moderate-to-vigorous physical activity.

**Table 2:** Results from three-level multiple linear regression models adjusted for BMI z-score, weekend/weekday and SES assessing difference in physical activity by pedometer wear status in adolescents (n = 390 boys; n = 502 girls; 14.5 ± 0.5 years) from November 2005 to July 2007 in Cambridgeshire and Suffolk, Eastern England.

	Boys β (95% CI) p value	Girls β (95% CI) p value
*Accelerometry (cpm)*		
Reference category (no pedometer)		
Wearing pedometer	− 9.4 (− 26.6 to 7.9) 0.30	5.1 (0.8 to 9.5) 0.02
Reference category (weekday)		
Weekend day	− 15.3 (− 28.2, − 2.3) 0.02	− 11.6 (− 14.7, − 8.6) < 0.001
Reference category (highest SES)		
Middle SES	− 1.9 (− 23.4, 19.5) 0.86	2.0 (− 3.3, 7.3) 0.465
Lowest SES	− 7.3 (− 32.5, 17.8) 0.57	5.9 (− 0.7, 12.6) 0.08
BMI z-score	− 1.2 (− 9.2, 6.8) 0.77	1.7 (− 0.4, 3.8) 0.11
Wald statistic for model chi2 (p value)	7.13 (0.21)	64.5 (< 0.001)

*MVPA (minutes/day)*		
Reference category (no pedometer)		
Wearing pedometer	− 4.7 (− 11.3, 1.9) 0.16	3.4 (− 0.8, 7.6) 0.11
Reference category (weekday)		
Weekend day	− 16.6 (− 21.3, − 11.9) < 0.001	− 10.0 (− 12.9, − 7.0) < 0.001
Reference category (lowest SES)		
Middle SES	5.9 (− 2.4, 14.2) 0.16	3.0 (− 2.1, 8.1) 0.24
Highest SES	0.7 (− 9.0, 10.4) 0.89	8.7 (2.3, 15.0) 0.008
BMI z-score	1.0 (− 2.0, 4.1) 0.51	1.5 (− 0.6, 3.5) 0.24
Wald statistic for model chi2 (p value)	53.1 (< 0.001)	53.9 (< 0.001)

cpm = counts per minute, MVPA = moderate-to-vigorous physical activity.
